# Frequencies of activated T cell populations increase in breast milk of HCMV-seropositive mothers during local HCMV reactivation

**DOI:** 10.3389/fimmu.2023.1258844

**Published:** 2024-01-03

**Authors:** Katrin Lazar, Graham Pawelec, Rangmar Goelz, Klaus Hamprecht, Kilian Wistuba-Hamprecht

**Affiliations:** ^1^Institute for Medical Virology and Epidemiology of Viral Diseases, University Hospital Tübingen, Tübingen, Germany; ^2^Department of Immunology, Interfaculty Institute for Cell Biology, University of Tübingen, Tübingen, Germany; ^3^Cancer Solutions Program, Health Sciences North Research Institute, Sudbury, ON, Canada; ^4^Department of Neonatology, University Children’s Hospital Tübingen, Tübingen, Germany; ^5^Section for Clinical Bioinformatics, Internal Medicine I, University Medical Center, Tübingen, Germany; ^6^M3 Research Center, University Medical Center, Tübingen, Germany

**Keywords:** monocytes, CD4+ T cells, CD8+ T cells, effector memory T cells, viral load, HCMV-specific T cells, virus transmission, breast feeding

## Abstract

**Background:**

Human cytomegalovirus (HCMV) can reactivate in the mammary gland during lactation and is shed into breast milk of nearly every HCMV-IgG-seropositive mother of a preterm infant. Dynamics of breast milk leukocytes during lactation, as well as blood leukocytes and the comparison between both in the context of HCMV reactivation is not well understood.

**Methods:**

Here, we present the BlooMil study that aimed at comparing changes of immune cells in blood and breast milk from HCMV-seropositive- vs -seronegative mothers, collected at four time ranges up to two months post-partum. Viral load was monitored by qPCR and nested PCR. Multiparameter flow cytometry was used to identify leukocyte subsets.

**Results:**

CD3^+^ T cell frequencies were found to increase rapidly in HCMV-seropositive mothers’ milk, while they remained unchanged in matched blood samples, and in both blood and breast milk of HCMV-seronegatives. The activation marker HLA-DR was more strongly expressed on CD4^+^ and CD8^+^ T cells in all breast milk samples than matched blood samples, but HCMV-seropositive mothers displayed a significant increase of HLA-DR^+^ CD4^+^ and HLA-DR^+^ CD8^+^ T cells during lactation. The CD4^+^/CD8^+^ T cell ratio was lower in breast milk of HCMV-seropositive mothers than in the blood. HCMV-specific CD8^+^ T cell frequencies (recognizing pp65 or IE1) were elevated in breast milk relative to blood, which might be due to clonal expansion of these cells during local HCMV reactivation. Breast milk contained very low frequencies of naïve T cells with no significant differences depending on serostatus.

**Conclusion:**

Taken together, we conclude that the distribution of breast milk leukocyte populations is different from blood leukocytes and may contribute to the decrease of breast milk viral load in the late phase of HCMV reactivation in the mammary gland.

## 1 Introduction

Breast milk is a highly immunologically active body fluid and is, therefore, not only important for the nutritional needs of the new-born infant, but also for protection against pathogens and establishment of a healthy gut microbiome (1–3). Breast milk cells (BMCs) are very heterogeneous, consisting of epithelial cells such as lactocytes and myoepithelial cells, stem cells, progenitor cells, monocytes, myeloid-derived suppressor cells (MDSCs), macrophages, granulocytes and lymphocytes (4). Colostrum has a high leukocyte count, constituting 13 - 70% of total breast milk cells (5). The total leukocyte count and also the CD16^-^ classical monocyte frequencies are reported to decrease over the lactation period (6). However, leukocyte frequencies may be increased in the mature milk of mothers with infections, and can account for >90% of all breast milk cells (7).

HIV, HTLV-1 and Zika (8) all employ breast milk as a mother-to-infant transmission route, a property also shared with human cytomegalovirus (HCMV) (9). HCMV belongs to the Herpesviridae and establishes lifelong persistence after primary infection. Sporadic reactivations or reactivations under immunosuppression are well described in the context of solid organ or stem cell transplant patients. Additionally, local HCMV reactivation can be studied in a healthy immunocompetent host in the mammary gland via detection of viral DNA in secreted milk without any invasive procedure (10). Up to 96% of HCMV IgG-seropositive mothers reactivate the virus in a unimodal, self-limited manner during breast feeding; viral loads with highly variable peak levels ranging from 10^3^ to 10^6^ copies/ml were reported (10). Transmission occurs in 37-42% of mature and preterm infants (11). If the infant is born prematurely with a gestational age under 32 weeks or a birthweight under 1500g, HCMV infection can lead to severe disease with sepsis-like symptoms (SLS) (12). Vertical HCMV transmission can be prevented by heat inactivation of breast milk especially in the case of at-risk preterm infants. This is the reason why breast milk should be inactivated for such preterm infants (13).

In an earlier preliminary study, we found first indications of an elevated T cell population in breast milk of HCMV-seropositive mothers (14). This observation is consistent with the known immune response of systemic active HCMV infection and is strong evidence that cellular immunity plays an important role in the local control of HCMV reactivation in the mammary gland and thus also in decreasing the viral load as observed in the unimodal course of reactivation (9, 15). To investigate this further, we designed and performed the here presented controlled prospective BlooMil study, where multiple leukocyte populations were monitored in breast milk and matched blood samples of HCMV-seropositive or –seronegative mothers up to two months postpartum, in order to gain insights into the influence of HCMV reactivation on monocyte and T cell kinetics.

## 2 Materials and methods

### 2.1 Study design

**Figure 1:**
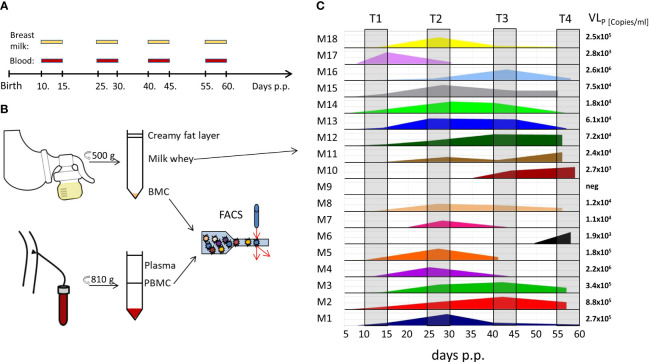
BlooMil study design and HCMV viral load in breast milk. **(A)** timeline of blood and breast milk sampling postpartum (p.p.). **(B)** Breast milk and blood sample processing for analysis. **(C)** viral load in milk whey of 18 HCMV-seropositive mothers measured by real time PCR. T1 – 10 to 15, T2 – 25 to 30, T3 – 40 to 45, and T4 – 55 to 60 days postpartum.

The BlooMil study design was reported earlier in detail (16). Briefly, mothers of mostly preterm infants from the Neonatology Department of the Children’s Hospital Tübingen were invited to participate and donate breast milk and EDTA-blood samples simultaneously at four defined time ranges after birth (T1 – 10 to 15, T2 – 25 to 30, T3 – 40 to 45, and T4 – 55 to 60 days postpartum) (**Figure 1A**). The first time point was chosen to detect the reactivation of HCMV in breast milk, which was observed around week one to two (17). The following time points were chosen to detect the peak value in breast milk and also the decline afterwards (17). 36 mothers participated in the BlooMil study, but eight had to be excluded due to lack of breast milk at later time ranges or transfer to regional hospitals. Finally, 18 HCMV-seropositive and 10 HCMV-seronegative mothers were analysed for breast milk viral loads and kinetics of breast milk (BMC) and peripheral blood mononuclear cell (PBMC) subset changes (**Table 1**). Mother 2 (red) was excluded from statistical analysis due to a HCMV primary infection during her pregnancy. Additionally, after the results of these phenotypic analysis confirmed our hypothesis, BMC and PBMC samples of four HCMV-seropositive HLA-A*02- positive mothers of preterm infants were analysed for the presence of HCMV pp65- or IE1-specific CD8^+^ T cells using a tetramer technique.

**Table 1:** Demographics of the BlooMil study population, as well as the extended BlooMil study cohort consisting of the four mothers used for tetramer analysis (at the end of the table).

Mother	HCMV-IgG	Age [years]	GA [weeks]	BW [g]	number of births	multi- births	color-coding of figures
1	pos	36	25 4/7	860	first	no	dark blue
2	pos	33	39 2/7	3100	second	no	red
3	pos	30	23 6/7	625	third	no	green
4	pos	33	24 2/7	580 + 780	first	twins	purple
5	pos	30	30 2/7	1165	second	no	orange
6	pos	33	28 5/7	1340	second	no	black
7	pos	28	33 0/7	2600	first	no	pink
8	pos	33	29 1/7	1190	first	no	beige
9	pos	39	38 1/7	3000	third	no	bright brown
10	pos	38	31 5/7	1540	second	no	dark red
11	pos	27	28 4/7	970 + 1170	first	twins	brown
12	pos	32	27 2/7	920 + 485 + 945	second	triplets	dark green
13	pos	49	27 6/7	880 + 1250	first	twins	blue
14	pos	36	34 2/7	2430	second	no	bright green
15	pos	31	31 4/7	1790	first	no	grey
16	pos	37	30 6/7	990 + 1410	first	twins	bright blue
17	pos	30	25 5/7	915	fourth	no	bright purple
18	pos	38	32 4/7	1970 + 2065	first	twins	yellow
mean	pos	34 ± 5	30 ± 4	1399			
19	neg	27	27 2/7	940	first	no	pastel green
20	neg	37	27 0/7	745	third	no	pastel blue
21	neg	31	26 2/7	515	second	no	pastel orange
22	neg	33	33 3/7	1990	first	no	pastel jade
23	neg	34	33 4/7	1800	first	no	pastel yellow
24	neg	31	29 5/7	1840	first	no	pastel grey
25	neg	33	24 5/7	550	second	no	pastel purple
26	neg	31	34 2/7	2100	first	no	pastel brown
27	neg	31	40 6/7	3470	first	no	pastel red
28	neg	34	30 3/7	1180	first	no	black
mean	neg	32 ± 3	31 ± 5	1351			
29	pos	33	27 5/7	920 + 905	3	twins	red
30	pos	29	27 4/7	940	3	no	green
31	pos	35	25 2/7	520	1	no	purple
32	pos	21	28 1/7	1120	1	no	blue

All mothers gave their informed written consent. Ethical approval was given by the Clinical Ethics Committee at the University Hospital Tübingen (project number: 804/2015BO2). Demographics of all mothers are given in **Table 1**.

### 2.2 Samples

For this prospective study, fresh breast milk samples on each day of measurement were prepared as described recently (16), except that after the first centrifugation step the cell pellet was washed twice with PBS. If the breast milk cell pellet had a reddish colour, which could be contamination by erythrocytes in the sample and therefore probably reflecting mastitis, the data points are shown as empty squares rather than round bullet points in the figures. Fresh EDTA-blood was diluted 1:1 with Hanks’ Balanced Salt solution (HBSS, Sigma-Aldrich, St. Louis, MO, USA) and PBMCs were isolated from the interface after FicoLite-H (Linaris blue, Dossenheim, Germany) gradient centrifugation (810g, 25 min, room temperature (RT)). Milk whey and plasma was investigated for the presence of HCMV-DNA using nested PCR (16). HCMV-DNA in milk whey was additionally detected using real time PCR to determine a quantitative readout as described before (16). After three washing steps of the cell compartment, samples were analysed directly via flow cytometry (**Figure 1B**). In addition, a biological control for the flow cytometry consisting of frozen PBMC aliquots derived from one leukapheresis was thawed on each day of measurement and stained in the same manner as all other samples.

### 2.3 Staining for flow cytometry analyses

All antibodies were obtained from Biolegend (San Diego, USA) or BD Biosciences (San Jose, USA) (see **Supplementary Table 1**) and were titrated before use. HCMV pp65 and IE1-peptide loaded tetramers were commercially accessed from the Tetramer Shop (**Supplementary Table 1**).

Up to 3x10^6^ fresh BMCs and 1x10^6^ fresh PBMCs were used for the staining procedure. Staining was for 20 min with antibodies in the dark at RT, followed by washing with 1 ml staining buffer (PBS with 2% FCS, 2 mM EDTA and 0.01% NaN_3_; termed PFEA) and centrifugation at 300g for 5 min at RT, unless stated otherwise. The first staining was performed with the dead cell marker ethidium monoazide bromide (EMA) and Fcγ receptors were blocked with GAMUNEX (human immunoglobulin; Grifols); the incubation step was performed under neon light. Another staining with an anti-CCR7 biotin antibody in PFEA followed. The samples were then incubated with streptavidin BV510 conjugate in PFEA. The last staining step included an antibody cocktail of anti-CD3, -CD8, -CD4, -CD14, -CD38, -CD45, -CD45RA, -CD56 and -HLA-DR antibodies. The stained samples were then immediately acquired on a LSR II (BD) flow cytometer.

To identify HLA-A*02-positive mothers for analysis of HCMV-specific CD8^+^ T cells using tetramers, 100 µl whole blood of HCMV-seropositive mothers was treated with 2 ml of 1x red blood cell lysis buffer (Biolegend) for 15 min in the dark at RT. After three washing steps with 4 ml PFEA, the cell pellet was stained with an anti-human HLA-A*02 PE antibody (Biolegend) and, after additional washing, acquired on a LSR II flow cytometer.

HCMV pp65 or IE1-specific CD8^+^ T cells were quantified in BMC and PBMC of HLA-A*02-positive mothers using HLA-A*0201 tetramers with the HCMV epitopes pp65 (495–504) NLVPMVATV and IE1 (316–324) VLEETSVML. After dead cell staining using EMA, tetramers were added to the samples diluted in PBS and incubated at 37°C for 15 min. After washing at RT, the samples were incubated with an antibody cocktail consisting of anti-CD3, -CD8, -CD4, -CD14, -CD45 antibodies on ice. Due to the low number of participants, no statistical analysis could be performed.

### 2.4 Flow cytometry gating

**Figure 2:**
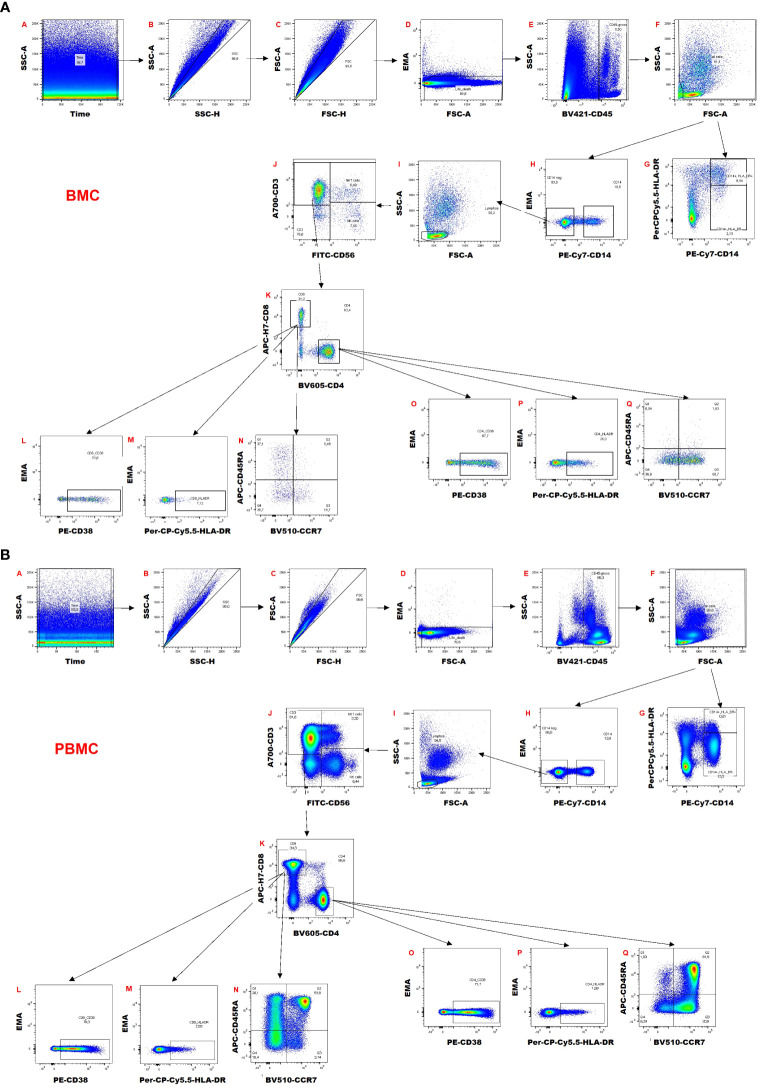
Gating strategy of **(A)** breast milk cells (BMC) and **(B)** peripheral blood mononuclear cells (PBMCs).

The gating strategy is shown in **Figure 2** for breast milk (**Figure 2A**) and blood samples (**Figure 2B**). Briefly, a time gate was used to exclude beginning and end of sample acquisition and to monitor any possible pressure fluctuations, if necessary, followed by exclusion of doublets and aggregates in the forward scatter (FSC) and sideward scatter (SSC) channels displaying height versus area signals. EMA-positive dead cells were excluded, and viable cells were gated on CD45^+^ cells to identify leukocytes. Next, a morphological gate was used to exclude debris and fat globules defining the lymphocyte population as the smallest population of cells of interest. Monocytic myeloid derived suppressor cells (M-MDSCs) were identified as CD14^+^ HLA-DR^-/dim^ cells. For the M-MDSCs, the same gates set in the corresponding blood sample were also applied for breast milk leukocytes. The CD45^+^ leukocyte population was also used to identify CD14^+^ Monocytes/Macrophages and CD14-negative cells. The latter were used to identify lymphocytes via a morphological gate and these were then further distinguished into CD3^+^ T cells, CD3^+^ CD56^+^ NKT-like cells and CD56^+^ NK cells. The CD3^+^ T cells were further divided into CD4^+^ and CD8^+^ subsets. Both subsets were investigated for HLA-DR and CD38 expression as well as for their memory subsets (naïve, central and effector memory and effector memory T cells re-expressing CD45RA (TEMRA) cells using expression/absence of CCR7 and CD45RA). The anti-CCR7 antibody was covalently coupled to biotin and detected via a streptavidin-BV510 conjugate. However, in the channel used to detect BV510, breast milk cells showed some autofluorescence. Therefore, the separation of effector and central memory T cell populations was more difficult to determine in breast milk samples than in blood samples. CCR7 separation in blood was reliable, hence the same gating was also used on breast milk T cells.

The gating for the tetramer analysis is shown in **Supplementary Figure 1**. A similar strategy as explained above was used. HCMV-specific CD8^+^ T cells were distinguished by an IE1 tetramer BV510 and APC double-positive population, and pp65 tetramer by a single positive APC population.

### 2.5 Statistics

For all statistical tests SPSS (version 25.0.0.1, IBM, Armonk, USA) was used. Mothers of the same cohort and sample type were analysed with the Friedman test (F) for the kinetics of each cell subset regarding continuity or change in frequencies over time. Dunn-Bonferroni *post-hoc* testing with Bonferroni correction (*post-hoc*) was used to determine statistically significant differences. Additionally, a linear mixed model (LMM) was used to analyze the data: The two factors HCMV serostatus and time as well as their interaction were included in the model as fixed effects and a repeated measurement analysis (REML) was carried out.

For the comparison of blood and breast milk samples within the HCMV seropositive or seronegative cohort at a certain time range (for example T1’s), the Wilcoxon signed-rank test with Bonferroni corrections for multiple testing was used (abbreviated as W). Mann-Whitney U testing with Bonferroni correction for multiple testing (abbreviated as MWU) was used to compare HCMV-seropositive with seronegative mothers’ cell subsets at any one-time range (for example T1’s).

Spearman correlation tests were used for calculating correlations between breast milk viral load and breast milk cells.

For the visualization of immune signatures, heatmaps of normalized frequencies of all cell subsets were generated with R (the R Foundation for Statistical Computing, version 4.0.3) using the complex heatmap package (18, 19). The dendrograms were calculated using maximum length apart for the row distance (based on Euclidean distance) and the ward.D2-approach for clustering.

## 3 Results

HCMV-seropositive and -seronegative mothers’ milk and blood was investigated at four different time points after birth (T1 – 10 to 15, T2 – 25 to 30, T3 – 40 to 45, and T4 – 55 to 60 days postpartum) to study the influence of local HCMV reactivation on the distribution of the different leukocyte subsets.

### 3.1 Demographics of the study cohorts and viral load during HCMV reactivation

The 18 HCMV-seropositive participants of the BlooMil study were 34 ± 5, while the 10 seronegatives were 32 ± 3 years old. Infants were born at a mean gestational age of 30 ± 4 weeks in the case of HCMV-seropositives (two term and 16 preterm infants) and of 31 ± 5 weeks in HCMV-seronegatives (one term and 9 preterm). The babies of HCMV-seropositive mothers averaged 1399 g birth weight, while the seronegatives weighed a mean of 1351 g (**Table 1**).

With the exception of mother 9, all seropositive mothers reactivated HCMV as shown by the presence of virus in breast milk (17 of 18, 94.4%) (**Figure 1C**). Mothers 6, 7, and 10 showed no reactivation at the first-time range, but later on at T4, T2 and T3, respectively. The calculated mean onset of viral shedding into breast milk was at 11.5 days p.p. Breast milk viral loads of the HCMV-seropositive mothers have already been analysed and published elsewhere using the same ID and color-coding (16) and ranged widely with peak levels between 10^3^ to 10^6^ (**Figure 1C**). With the knowledge of the initial viral reactivation process (**Figure 1C**) changes within the composition of the cellular signatures in milk and blood can be attributed to the presence of the virus in milk.

All HCMV-IgG-seronegative mothers were negative for HCMV DNA in the longitudinal investigation of breast milk and blood samples.

### 3.2 Monocytes/macrophages and M-MDSCs

**Figure 3:**
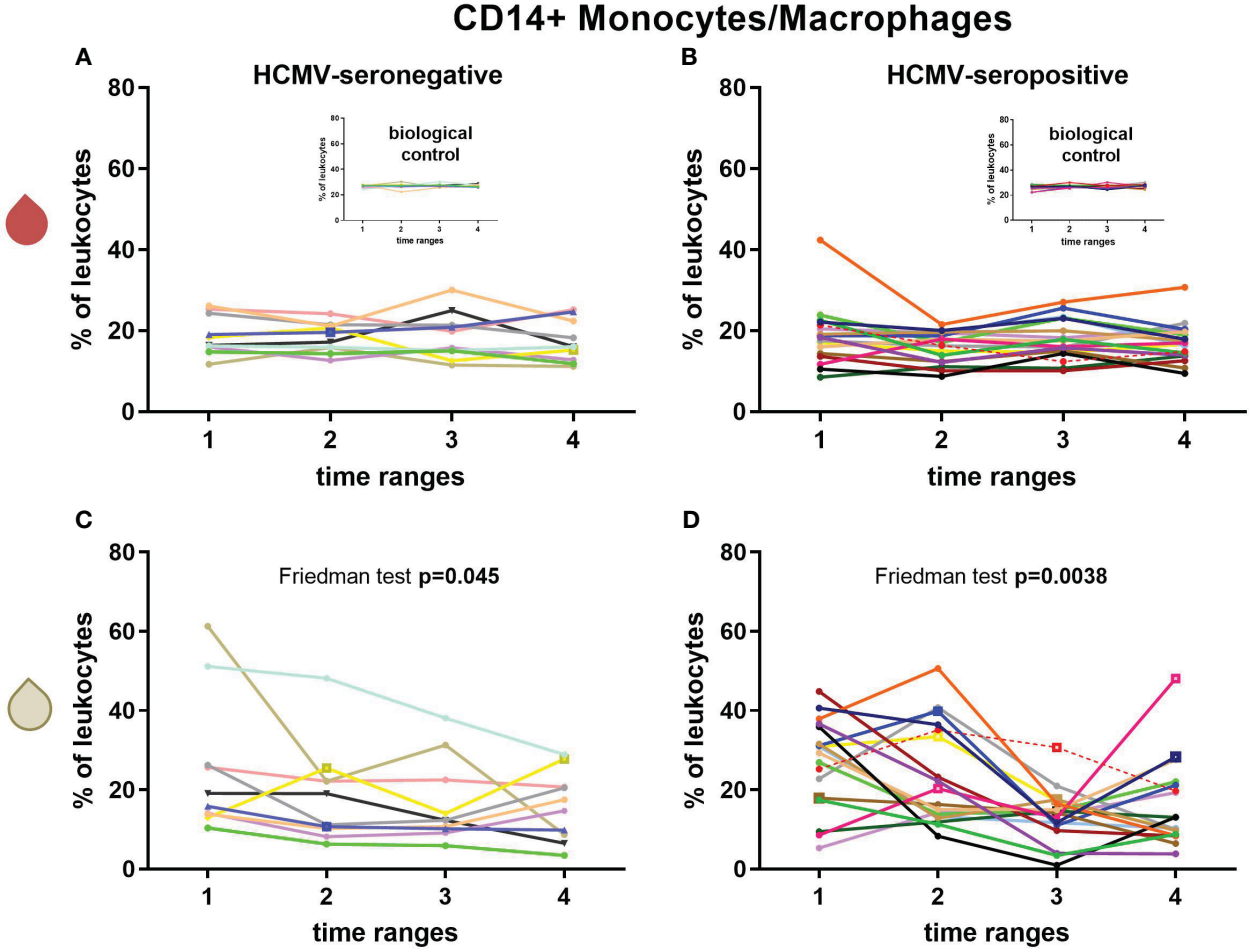
Longitudinal courses of CD14+ monocyte/macrophage frequencies in blood (red droplet) of HCMV-seronegative **(A)** and seropositive **(B)** mothers and in breast milk (white droplet) of HCMV-seronegative **(C)** and positive **(D)** mothers. The inserts in **(A, B)** show the biological control over all experimental days. Individual color-coding was applied as shown in **Table 1**. Mother 2 (red) was excluded from statistical analysis due to an HCMV primary infection during pregnancy. Empty squares as data points indicate probable mastitis at this time point. Time ranges were T1 – 10 to 15, T2 – 25 to 30, T3 – 40 to 45, and T4 – 55 to 60 days postpartum.

CD14^+^ monocyte/macrophage frequencies in blood showed no significant changes over time (**Figures 3A, B**, **Supplementary Table 2**). In breast milk of HCMV-seronegative (Friedman test (F): p=0.045, **Figure 3C**) or HCMV-seropositive (F: p=0.004, *post-hoc*: T1-T3 p=0.013, T1-T4 p=0.032, **Figure 3D**) mothers, the CD14^+^ monocyte/macrophage frequency significantly decreased over time.

M-MDSC frequencies were around 1-2% of leukocytes at the later time ranges (**Supplementary Table 2**) which is very low in breast milk. A significant decrease in the frequency of M-MDSCs over time in breast milk of HCMV-seropositive but not in seronegative mothers was observed (F: p=0.0088, *post-hoc*: T1 to T3 p=0.047) (**Supplementary Table 2**).

### 3.3 T cells and NK cells

**Figure 4:**
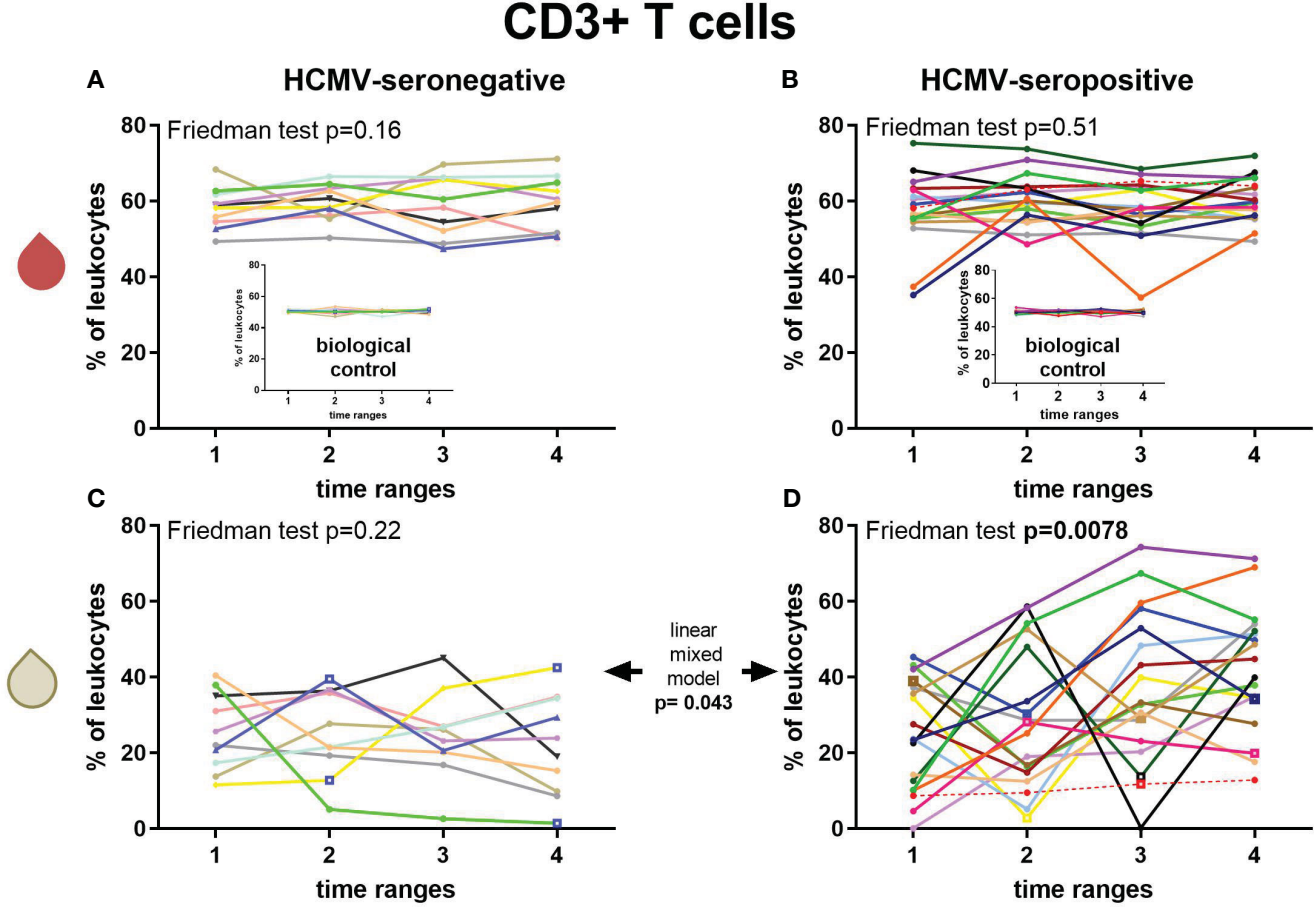
Longitudinal courses of CD3+ T cell frequencies in blood (red droplet) of HCMV-seronegative **(A)** and seropositive **(B)** mothers and in breast milk (white droplet) of HCMV-seronegative **(C)** and positive **(D)** mothers. The inserts in **(A, B)** show the biological control over all experimental days. Individual color-coding was applied as shown in **Table 1**. Mother 2 (red) was excluded from statistical analysis due to an HCMV primary infection during pregnancy. Empty squares as data points indicate probable mastitis at this time point. Time ranges were T1 – 10 to 15, T2 – 25 to 30, T3 – 40 to 45, and T4 – 55 to 60 days postpartum.

Longitudinal CD3^+^ T cell frequencies in breast milk and blood of HCMV-seropositive and seronegative mothers were measured (**Figures 4A–D**). Breast milk CD3^+^ T cells of HCMV-seropositive mothers showed a significant increase by 18.6% in mean frequencies (F: p=0.0078, *post-hoc* T1 to T4 p=0.032, T2 to T4 p=0.047, **Figure 4D**), whereas HCMV-seronegative frequencies did not significantly change over time (**Figure 4C**). Thus, a significant difference of HCMV-seropositive vs seronegative mothers’ breast milk T cell frequencies was observed over the time course (linear mixed model (LMM): p=0.043). There also was a significant difference of T cell frequencies in breast milk of HCMV-seropositives and seronegatives at T4 (MWU: 0.0057). However, no significant correlations of T cell frequencies with breast milk viral loads were found (despite T3 with a moderate correlation, ρ=0.49). CD3^+^ T cell frequencies in blood of all BlooMil study participants remained relatively constant over the observation period (**Figures 4A, B**).

While CD4^+^ T cell frequencies amongst all CD3^+^ T cells in blood of HCMV-seropositive and -negative mothers were similar, breast milk frequencies were lower in HCMV-seropositive than in seronegative mothers, especially at T4 (Mann-Whitney U test (MWU): p<0.0001, **Supplementary Table 2**). Additionally, only seropositive mothers displayed significantly lower CD4^+^ T cell frequencies of all T cells in breast milk than in the matched blood sample (W for all time range pairs: p<0.008, **Supplementary Figure 2A**).

Kinetics of CD8^+^ T cell frequencies of all T cells in breast milk showed slight increases over time in HCMV-seropositive mothers but slight decreases in seronegative mothers (LMM: p=0.028, **Supplementary Table 2**). In general, breast milk of HCMV-seropositive mothers had significantly higher CD8^+^ T cell frequencies than in seronegatives at almost all time ranges (MWU: T2’s=0.019, T3’s=0.037, T4’s=0.00026, **Supplementary Figure 2B**).

**Figure 5:**
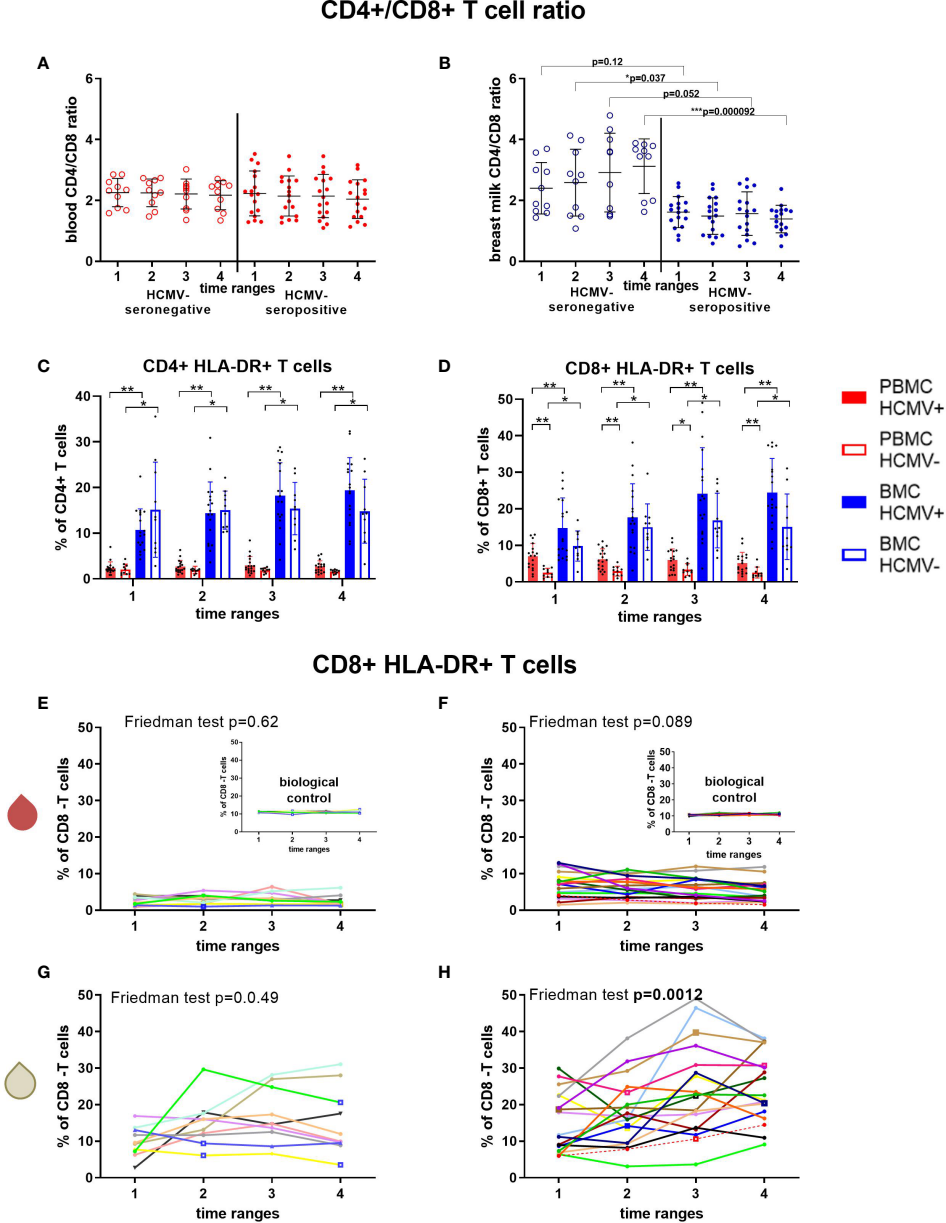
T cell subsets of HCMV seropositive and seronegative mothers. CD4^+^/CD8^+^ T cell ratio in blood **(A)** and breast milk **(B)** are shown. The activation marker HLA-DR was investigated on CD4+ **(C)** and CD8+ T cells **(D)**. Longitudinal analysis of CD8+ HLA-DR+ T cells in blood (red droplet) of HCMV-seronegative **(E)** and seropositives **(F)**, as well as in breast milk (white droplet) of HCMV seronegatives **(G)** and seroposiitves **(H)** are shown. The inserts in **(E, F)** show the biological control over all experimental days. Individual color-coding was applied as shown in **Table 1**. Mother 2 (red) was excluded from statistical analysis due to an HCMV primary infection during pregnancy. Empty squares as data points indicate probable mastitis at this time point. Statistical significances were indicated as followed: *p<0.05, **p<0.01, ***p<0.001.

The CD4/CD8 ratio was used to determine the proportion of cytotoxic T cell frequencies in relation to T helper cells. While the blood ratio remained constant over time and serostatus (**Figure 5A**), the ratio in breast milk of HCMV-seronegative mothers had significantly higher values than HCMV-seropositive mothers (MWU: T2’s p=0.037, T4’s p=0.000092; **Figure 5B**) and increased over time.

CD38 and HLA-DR surface expression was investigated as different activation markers for T cells (**Figure 5**, **Supplementary Figures 2C, D**). CD8^+^ HLA-DR frequencies in blood were higher in HCMV-seropositive than -seronegative mothers (**Figures 5D–F**), but breast milk cells had significantly higher frequencies of HLA-DR^+^ cells than blood regardless of serostatus (**Figures 5C, D, G, H**). However, only HCMV-seropositive mothers displayed an increase in the frequency of HLA-DR-expressing CD4^+^ and CD8^+^ T cells in breast milk over time (F: p≤0.0012, **Supplementary Table 2**, **Figure 5H**). CD38^+^ CD8^+^ T cell frequencies showed a tendency to increase only in HCMV-seropositive mothers´ breast milk (F: p=0.083, **Supplementary Table 2**, **Supplementary Figure 2D**). In blood, only HCMV seropositive mothers showed a significant decrease in CD8^+^ (F: p<0.001) and CD4^+^ (F: p=0.006, **Supplementary Table 2**) T cells expressing CD38, which might indicate a migration of activated HCMV-specific T cells to the mammary gland (20).

### 3.4 T cell differentiation profile

**Figure 6:**
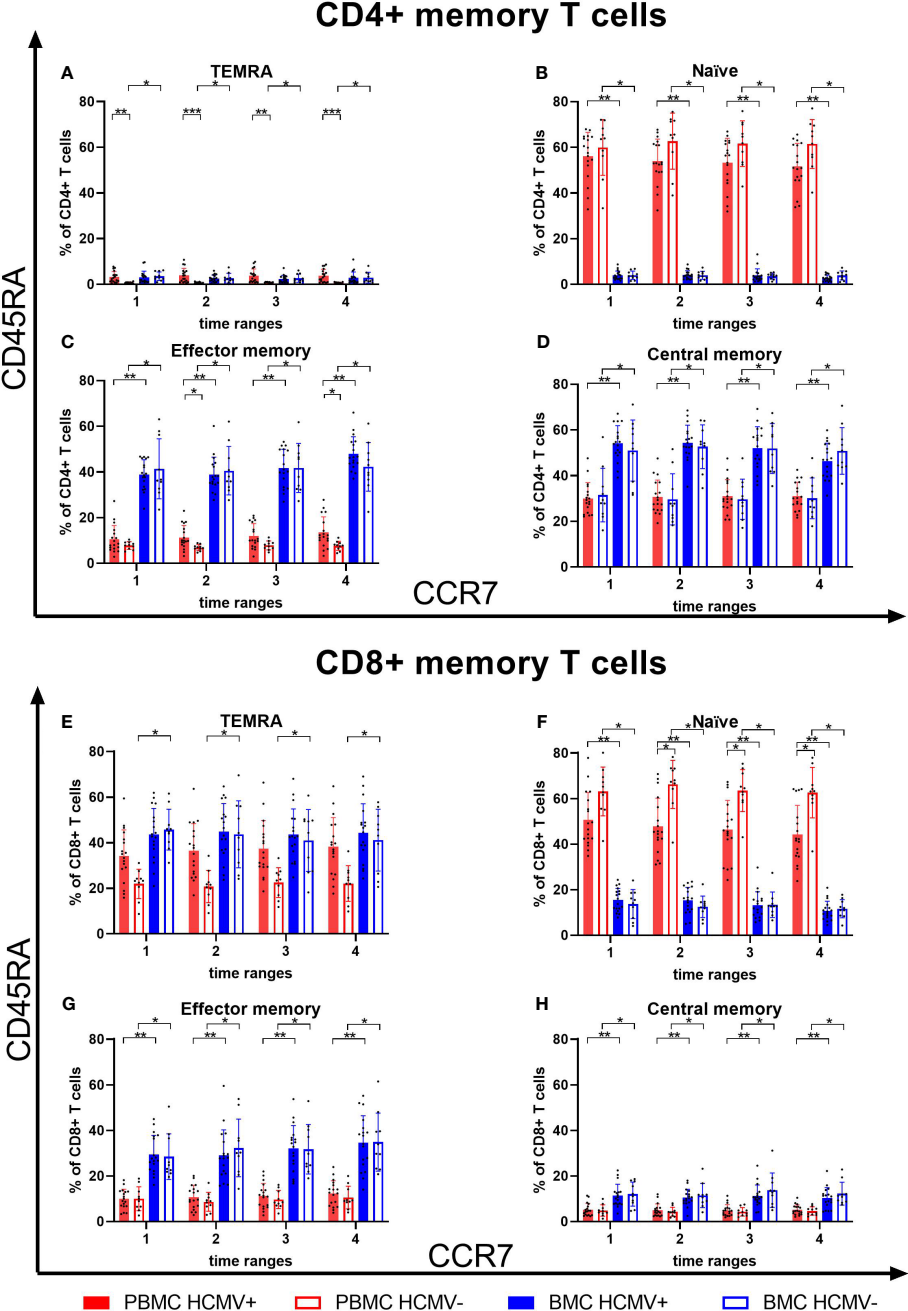
T cell memory subsets in breast milk and blood. CD4+ TEMRA **(A)**, naïve **(B)**, effector memory **(C)** and central memory **(D)** T cells of blood and breast milk from HCMV seropositive and seronegative mothers. Additionally, CD8+ TEMRA **(E)**, naïve **(F)**, effector memory **(G)** and central memory **(H)** T cells of blood and breast milk from HCMV seropositive and seronegative mothers were shown. Memory subsets were determined using CCR7 and CD45RA. Time ranges were T1 – 10 to 15, T2 – 25 to 30, T3 – 40 to 45, and T4 – 55 to 60 days postpartum. Statistical significances were indicated as followed: *p<0.05, **p<0.01, ***p<0.001.

The CD4^+^ T cell compartment in peripheral blood was dominated by a naïve phenotype, while in breast milk almost no naïve CD4^+^ T cells were present (**Figures 6A–D**). The latter was mainly dominated by central (CM) and effector memory (EM) CD4^+^ T cells, but there were no differences between HCMV-seropositive and -negative mothers. However, frequencies of CD4^+^ EM T cells in breast milk were significantly higher than in blood. For CD8^+^ T cells, the main subsets in blood were naïve and TEMRA cells (**Figures 6E, F**). HCMV-seropositive mothers had significantly lower proportions of naïve T cells than seronegative mothers in blood (**Figure 6F**). The breast milk CD8^+^ T cell compartment was composed of very low frequencies of naïve T cells (**Figure 6F**), but significantly higher frequencies of CM and EM T cells were found in breast milk compared to blood (**Figures 6G, H**). The main CD8^+^ subsets in breast milk were TEMRA and effector memory T cells in contrast to blood (**Figures 6E–H**).

### 3.5 NK cells

NK cell frequencies were less dominant at around 5% of all lymphocytes in breast milk and twice that in blood, regardless of the serostatus (**Supplementary Table 2**, **Supplementary Figure 2E**). No notable differences were found over time.

### 3.6 CD56^+^ T cells

Mean CD56^+^ T cell frequencies, including classical CD8^+^, γδ, and NKT-like cells, were around 3-5% in breast milk and in blood and also showed no major changes over time (**Supplementary Table 2**, **Supplementary Figure 2F**).

### 3.7 Unsupervised profiling of the composition of breast milk leukocytes

**Figure 7:**
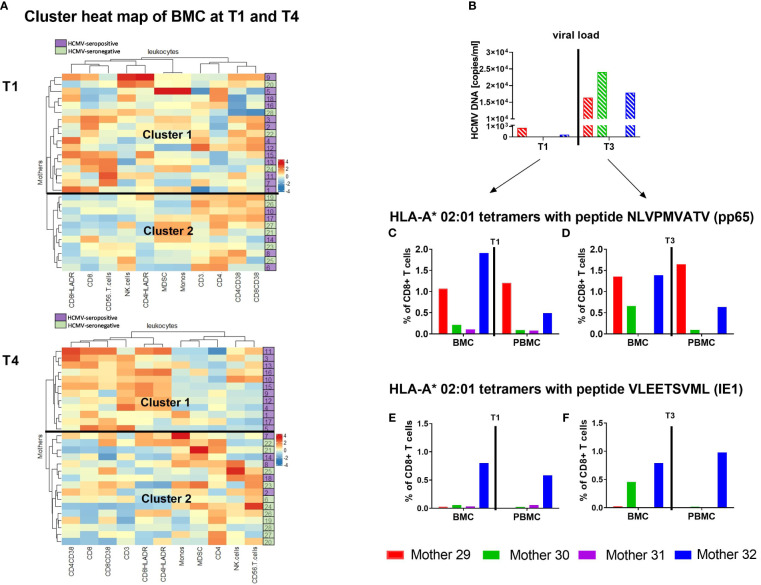
Heatmap of T cells in breast milk and HCMV-specific T cells. Clustering of breast milk cells (BMC) such as Monocytes, CD3+, CD4+ and CD8+ T cells etc. of HCMV-seropositive (purple) and seronegative (green) mothers at T1 and T4 **(A)** using the complex heatmap package (18, 19) is presented. It was calculated using maximum length apart for the row distance and the ward.D2-approach for clustering. Additionally, four mothers were analysed for their HCMV-specific T cell frequency using HLA-A*02 tetramer analysis (pp65 NLVPMVATV and IE1 VLEETSVML). Viral load in milk whey **(B)**, as well as the pp65-specific T cell frequencies of those mothers at T1 – 10 to 15 **(C)** and T3 – 40 to 45 days postpartum **(D)** are shown. Furthermore, IE1-specific T cell frequencies at T1 – 10 to 15 **(E)** and T3 – 40 to 45 days postpartum **(F)** are presented.

Clustering of the immune cell signature data of breast milk leukocytes from HCMV-seronegative-vs-seropositive mothers at T1 and T4 (T1 and T4 were used due to the increase in T cells and therefore highest cell changes expected) identified two main groups. Using the subsets of myeloid cells and all other leukocyte subgroups, the clustering at T1 resulted in no clear groups of seronegative and seropositive mothers. However, the clustering of T4 showed 100% of the seropositives in cluster 1 and 100% of the seronegative mothers in cluster 2, making up 62.5% of the whole cluster 2 (**Figure 7A**). The 5 seropositive mothers in the seronegative cluster 2 showed medium viral loads with 10^4^-10^5^ copies/ml and displayed no other clinical signs known to us which might indicate a clustering into the opposite group. When only CD3, CD4 and CD8 T cells were used for analysis, cluster 2 at T4 consisted of 81.8% seronegative mothers, while T1 showed again less specific clustering (**Supplementary Figures 3A, B**).

### 3.8 HCMV-specific T cells

To find a reason for the unimodal kinetics of HCMV reactivation and its presence in breast milk, HCMV-specific CD8^+^ T cells were investigated in closer detail. HCMV-seropositive and HLA-A*02-positive mothers were investigated for HCMV-specific CD8^+^ T cells in breast milk and blood at T1 (before/start of reactivation) and T3 (at/after peak viral load). The tetramer analysis for pp65 peptide (NLVPMVATV) recognition showed higher frequencies in breast milk than in blood for some mothers (**Figures 7C, D**). The mothers with no viral load in breast milk at T1 showed lower frequencies (**Figure 7B**). However, the frequencies of HCMV-specific T cells were not directly related to the viral load in our small cohort of mothers as soon as reactivation was observed. When comparing her own two time ranges, the mother with the highest viral load at T3 (mother 30) had higher frequencies of pp65- and IE1-specific CD8^+^ T cells in comparison to the earlier time range and the corresponding blood sample (**Figures 7C–F**). These data show, albeit unfortunately anecdotally due to the small number of cases, that the previously described associations of HCMV with T cell populations in breast milk are substantiated by the tendentially higher abundance of HCMV-specific CD8^+^ T cells in breast milk compared to blood.

## 4 Discussion

The observed differences in HCMV-seropositive in comparison to HCMV-seronegative mothers’ leukocyte subpopulations in breast milk presented here implies a cell-mediated immune reaction to the local reactivation of HCMV in the mammary gland. In the present study, HCMV-seropositive mothers showed an increase in T cells, especially in activated CD4^+^ and CD8^+^ T cells while the viral load was decreasing in most mothers after T2 or T3. This increase was mostly preceded by an increase of the lymphocyte attractant CXCL10 in the same cohort, as published earlier while analysing cytokines in breast milk whey (21). This finding is consistent with the here observed increase in the CD3^+^ T cell frequency by 18.6% over the lactation period from T1 to T4. Hassiotou et al. (7) reported an increase in breast milk leukocytes when mothers had an acute infection (independent of the responsible pathogen), but they did not include HCMV reactivation in their analysis, and the increases they recorded occurred within days. Still, in our study, the increase of T cells did not correlate strongly with breast milk viral load, probably due to the later onset of adaptive immunity to HCMV reactivation.

The design of the BlooMil study included investigation of CD4^+^ and CD8^+^ T cells in blood and matched breast milk. The CD4^+^ and CD8^+^ T cell frequencies in colostrum were reported earlier as more equivalent to each other compared to blood, but again, without consideration of HCMV serostatus (22, 23). In our study, the CD4/CD8 ratio of HCMV-seropositive mothers was actually lower in breast milk than in blood, due to a higher CD8^+^ T cell frequency in breast milk than blood. This finding indicates a shift to a milieu which could directly kill and eliminate virus-infected cells. Additionally, we found that these breast milk CD8^+^ T cells and the CD4^+^ T cells showed higher frequencies of the activation marker HLA-DR than in blood, confirming earlier studies (22, 24, 25). In our cohort of HCMV reactivating mothers, the activated T cell frequency increased significantly over time. This might be indicating selective migration of activated (CD8^+^) T cells into the mammary gland. The question of whether these cytotoxic T cells are also HCMV-specific was addressed in our extended BlooMil study cohort with four additional mothers. When the virus reactivated in breast milk, more HCMV-specific T cells were found in breast milk than in blood. Published multimer studies of HCMV-specific T cells in breast milk are very rare. Sabbaj et al. (24) analyzed one HLA-B*07:02-positive mother and additionally performed ELISPOT analysis, also finding slightly higher frequencies in breast milk than in blood. Moylan et al. (26), who used intracellular cytokine staining after peptide stimulation, also reported higher frequencies of HCMV-specific T cells in breast milk than blood. In our study cohort of four mothers, pp65-specific T cells were more often seen and, additionally, at higher frequencies than IE1-specific T cells. Kern et al. (27) reported that HLA-A*02-positive mothers more often had a higher response against pp65 than IE1.

Regarding the immune cell differentiation profile in breast milk of our study cohort, we showed a more activated phenotype than in blood, independent of HCMV-serostatus. We found very low frequencies of naïve T cells in all breast milk samples. Naïve T cells were reported to be rare in breast milk in an earlier study (24). Especially effector memory cells were found in breast milk, which is similar to other mucosal sites and implies the presence of a ready-to-act T cell subpopulation in breast milk.

T cells in breast milk might also consist of tissue resident memory T cells (T_RM_). T_RM_ cells are residing in tissue and can control HCMV reactivation events in the peripheral tissue quickly without the support of circulating T cells (28). To the best of our knowledge T_RM_ should also reside in the mammary gland but are mostly discussed in the context of breast cancer. If these cells are involved in anti-HCMV immunity, it is likely that they might also be detectable in breast milk. T_RM_ cells express CCR7 at a lower level (29), which might be one of the reasons that our memory subpopulations in breast milk were not as easy to define as in blood samples. However, such analyses remain for future studies because typical T_RM_ markers were not included here.

NK cells usually play a major role in the early response against HCMV infection (30). However, in our study, the frequency of CD56^+^ cells did not associate with HCMV serostatus and was lower in breast milk than in blood. This might suggest that NK cells are not specifically guided into the mammary gland, resulting in low frequencies in milk, and thus they seem to play a less important role in HCMV load reduction during lactation.

M-MDSCs as prominent regulators of T cell activity (31, 32) may reflect a potentially immunosuppressive milieu in breast milk. However, their frequencies in breast milk were very low suggesting any immunosurveillance by T cells would be less likely to be suppressed. There is an early study describing M-MDSCs as a minor MDSC population in breast milk and suggesting T cell suppression mainly by granulocytic MDSCs (33). However, that study also failed to consider HCMV. Unfortunately, we were unable to assess granulocytic MDSCs in the present study, but the immune signature kinetics delineated here do not suggest a prominent involvement of suppressor cells. However, the role of granulocytic MDSC could not be clarified and needs additional research in the context of maternal HCMV-serostatus.

To the best of our knowledge, there is no published literature on why and how HCMV is reactivating in the mammary gland. The here presented study provides insights regarding immunosurveillance during the phase of decreasing viral load in the second half of the reactivation process. Our favoured hypothesis to explain the complete unimodal, self-limited reactivation course of HCMV is mainly based on the knowledge gained from antibody, cytokine and leukocyte studies within the BlooMil study cohort (16, 21) and previous studies on HCMV reactivation in breast milk (**Supplementary Figure 4**). Hormones, cytokines and monocytes might substantially contribute to triggering reactivation of HCMV (**Supplementary Figure 4**). Especially monocytes, which are known to be latently infected with HCMV, could play a role here, because by migrating into tissue and differentiating into macrophages, HCMV can be reactivated, especially in the prevailing inflammatory milieu (21, 34, 35). Breast milk CD14^+^ macrophages were found to be positive for HCMV IE1 DNA (15). The slightly greater decrease of monocytes/macrophages in seropositive mothers in the present study could indicate that infected monocytes have been eliminated by T cells. However, such interactions/dependencies need to be addressed in future studies ideally comprising larger cohorts. In general, increasing the little data on local HCMV reactivation should be addressed in future research to better understand why for example not every mother is reactivating the virus and how the reactivation could be controlled. This is especially relevant for preterm infants as the majority of the immune activity compartment of breast milk is currently destroyed by pasteurization (36). To further develop our hypothesis (**Supplementary Figure 4**), a unimodal course of viral load is seen in almost all our study mothers, as published earlier (16, 37) and seems to be the normal pattern of reactivation. The decrease of the viral load in breast milk is most likely due to the activation of the immune system by the virus. Neutralizing antibodies and leukocytes such as HCMV-specific CD8^+^ T cells, are likely to play a major role in this context. We have also been able to show that HCMV-specific IgG titers of our HCMV-seropositive BlooMil study mothers were very low in breast milk compared to blood (16). Therefore, additional support from T cell killing infected breast tissue might be essential to reduce the viral load in the decreasing stage of reactivation (**Supplementary Figure 4**).

There are clearly some limitations of our study. The cohort of our BlooMil study was relatively small, because mothers of preterm infants have low amounts of breast milk to spare and the nutrition of the infant always has priority. This applies especially for colostrum samples; one reason why this study has the earliest starting time point at day 10. Furthermore, breast milk itself showed high inter-individual variability. Circadian effects can also influence the breast milk leukocyte subsets (38–40). Actual HCMV transmission events could not be documented because the breast milk was pasteurized before these at-risk infants received it. Another limitation of this study is the use of different cell purification protocols for blood (gradient isolation) and breast milk (centrifugation) resulting in the inclusion of granulocytic cells in analyses of the latter but not the former.

In summary, we posit that although the humoral IgG immune response including HCMV-specific neutralizing capacity might contribute to viral decrease, cellular immunity is also implicated as an important player: namely, the increase of activated (HCMV-specific) T cells could possibly contribute to the observed decreased viral load in the late stage of the unimodal reactivation by killing infected cells in the mammary gland. Furthermore, and despite the fact that infants of HCMV-seropositive mothers can become infected with HCMV via breast milk, they also receive more T cells than babies of HCMV-seronegative mothers. These seem to mainly consist of active, effector memory CD8^+^ T cells. A recent study reported elevated CD8^+^ T cell counts in breast milk and suggested that even after digestion, the dialyzable leukocyte extract (DLE) of CD8^+^ effector memory T cells (but not of other memory subsets) may still support the generation of antigen-specific cellular immunity passively transferred from the mother to the infant (41). Whether this might be a novel route for passive transfer of immunity needs to be clarified in future studies. However, in our study, an elevated frequency of HCMV-specific T cells was found, but presumably T cells in general increase in breast milk of HCMV-seropositive mothers. It is clear that infants receive more maternal T cells, especially CD8^+^ T cells, from HCMV-seropositive than infants of seronegative mothers. Animal models showed that such cells could remain intact and were found to localize in Peyer’s patches of murine pups (42, 43). If this is similar in humans, or if the lysed extracts of T cells do have an impact on the infant´s developing immune system, this would be of importance (44, 45), and needs to be addressed in future experiments regarding outcomes of different childhood diseases in infants of HCMV-seropositive mothers. However, the leukocyte content in breast milk might also merely mirror the inflammatory milieu in the mammary gland and might not be of importance for the development of the infant’s immune system after all.

In conclusion, this hypothesis-generating study i) supports the hypothesis of a local HCMV reactivation in breastfeeding mothers, ii) associates memory immune responses with the latter and iii) presents breast milk as a compartment to identify such immune responses and thus also as a medium transmitting both virus and anti-viral immunity from mother to child.

## Data availability statement

The raw data supporting the conclusions of this article will be made available by the authors, without undue reservation.

## Ethics statement

The studies involving humans were approved by Clinical Ethics Committee at the University Hospital Tübingen (Project number: 804/2015BO2). The studies were conducted in accordance with the local legislation and institutional requirements. The participants provided their written informed consent to participate in this study.

## Author contributions

KL: Data curation, Formal analysis, Investigation, Methodology, Visualization, Writing – original draft, Writing – review & editing. GP: Conceptualization, Methodology, Validation, Writing – review & editing. RG: Conceptualization, Methodology, Validation, Writing – review & editing. KH: Conceptualization, Data curation, Formal analysis, Investigation, Methodology, Resources, Supervision, Validation, Visualization, Writing – original draft, Writing – review & editing, Funding acquisition, Project administration. KW-H: Conceptualization, Data curation, Formal analysis, Investigation, Methodology, Resources, Supervision, Validation, Visualization, Writing – original draft, Writing – review & editing.
