# Supplementary material for: Frequencies of activated T cell populations increase in breast milk of HCMV-seropositive mothers during local HCMV reactivation
Source: Front Immunol. 2024 Jan 3;14:1258844. doi: 10.3389/fimmu.2023.1258844 (PMC10792025; doi:10.3389/fimmu.2023.1258844)
Supplement: Supplementary file 1 [file DataSheet_1.docx]

Supplementary Material

Frequencies of activated T cell populations increase in breast milk of HCMV-seropositive mothers during local HCMV reactivation

Katrin Lazar^1^, Graham Pawelec^2,3,^ Rangmar Goelz^4^, Klaus Hamprecht^1^, Kilian Wistuba-Hamprecht*^2,5,6^

^1^Institute for Medical Virology and Epidemiology of Viral Diseases, University Hospital Tübingen, 72076 Tübingen, Germany

^2^ Department of Immunology, Interfaculty Institute for Cell Biology, University of Tübingen, Tübingen

^3^ Cancer Solutions Program, Health Sciences North Research Institute, Sudbury, ON, Canada

^4^ Department of Neonatology, University Children’s Hospital Tübingen, 72076 Tübingen, Germany

^5^ Section for Clinical Bioinformatics, Internal Medicine I, University Medical Center, Tübingen, Germany

^6^ M3 Research Center, University Medical Center Tübingen, Germany

*** Correspondence:**Kilian Wistuba-Hamprecht
[Kilian.Wistuba-Hamprecht@uni-tuebingen.de](mailto:Kilian.Wistuba-Hamprecht@uni-tuebingen.de)

Katrin Lazar

Katrin.Lazar@med.uni-tuebingen.de


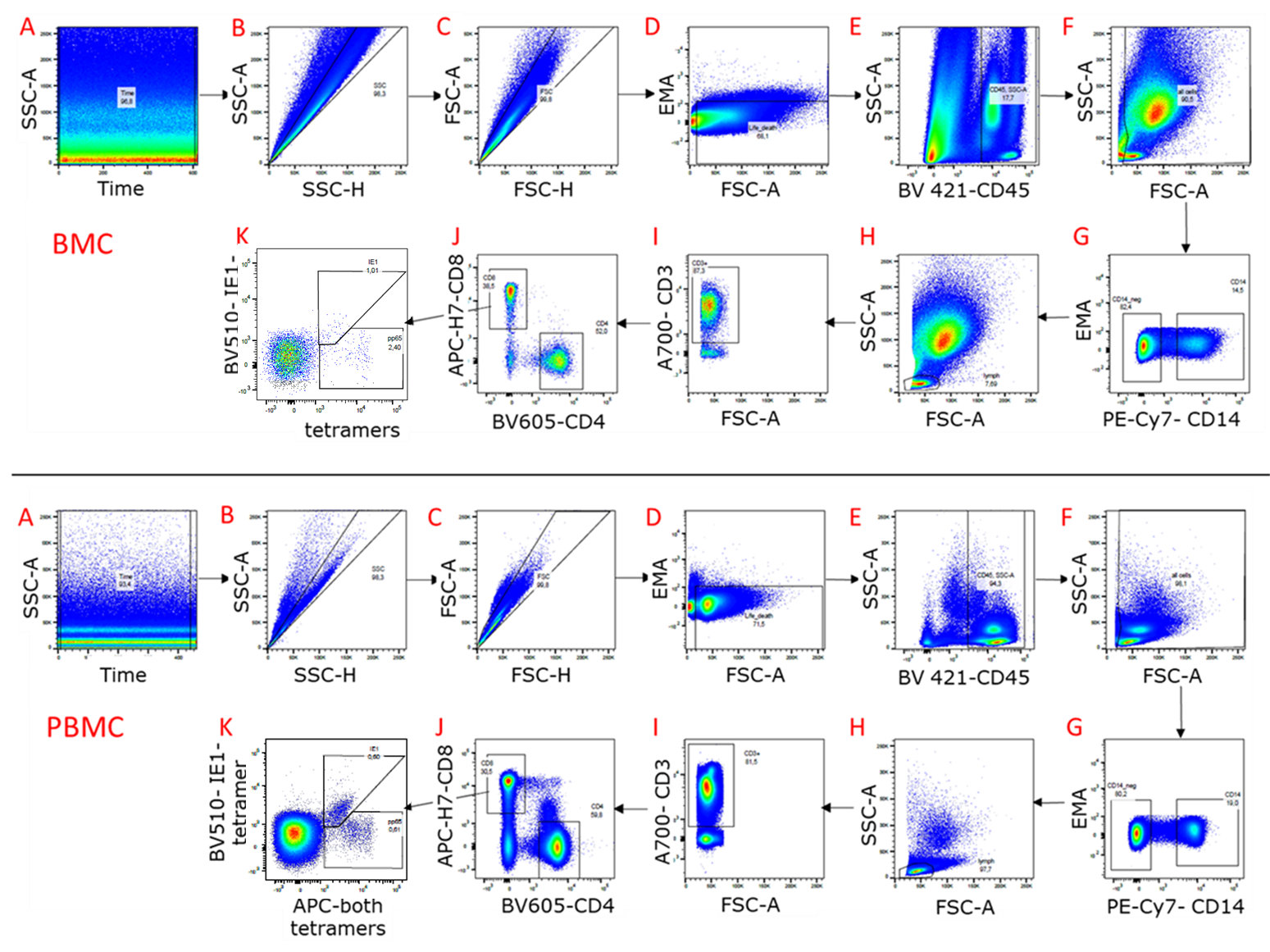


**Supplementary Figure 1.** Gating strategy of the tetramer analysis of breast milk cells (BMC) and PBMCs.

**Supplementary Figure 2.** Lymphocyte subset frequencies in breast milk and blood of HCMV seropositive and negative mothers. Time ranges were T1 – 10 to 15, T2 – 25 to 30, T3 – 40 to 45, and T4 – 55 to 60 days postpartum. Statistical significances were indicated as followed: *p<0.05, **p<0.01, ***p<0.001.


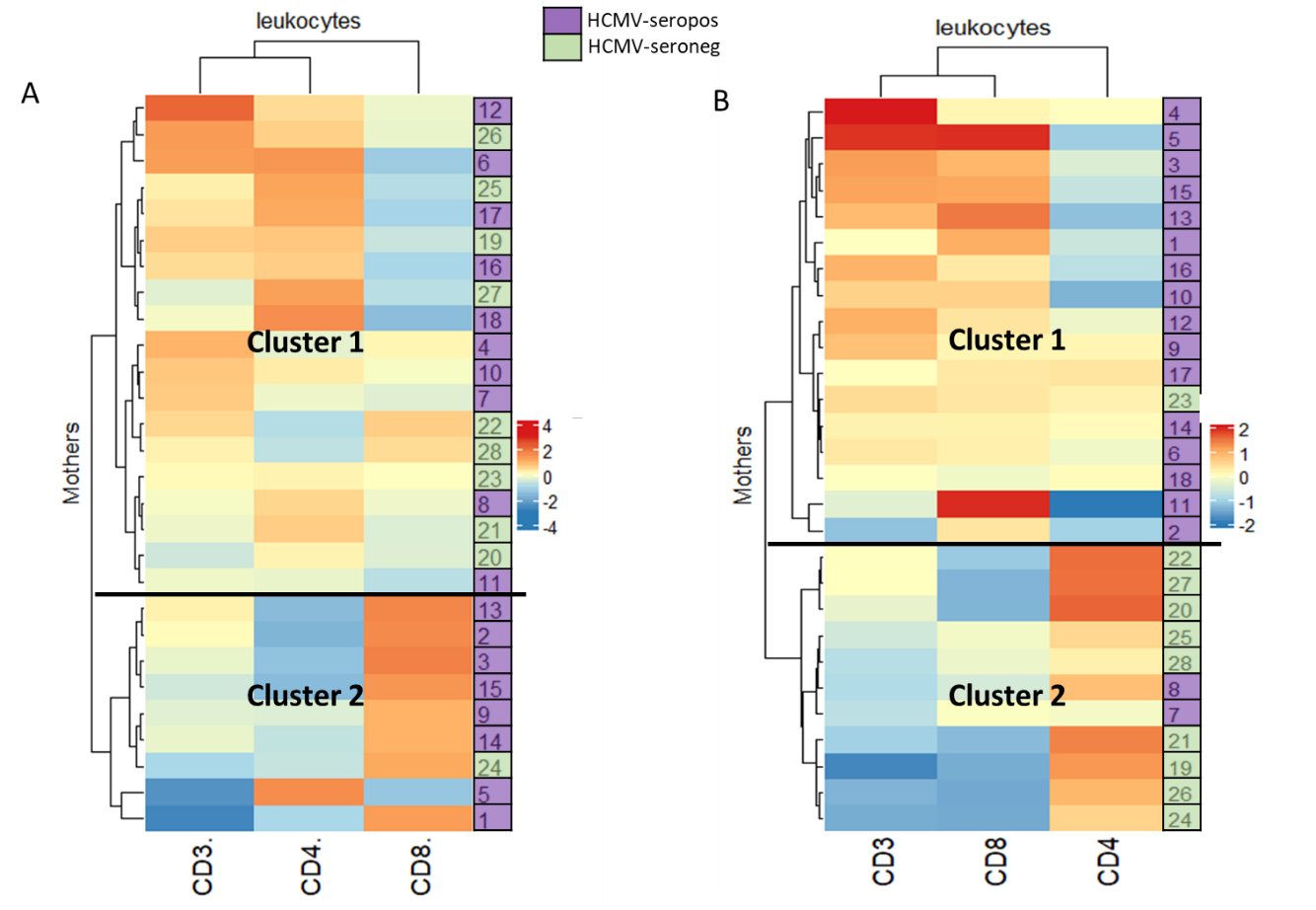


**Supplementary Figure 3.** Heatmap of breast milk leukocytes at A T1 and B T4 of HCMV-seronegative (outlined in green) and seropositive mothers.


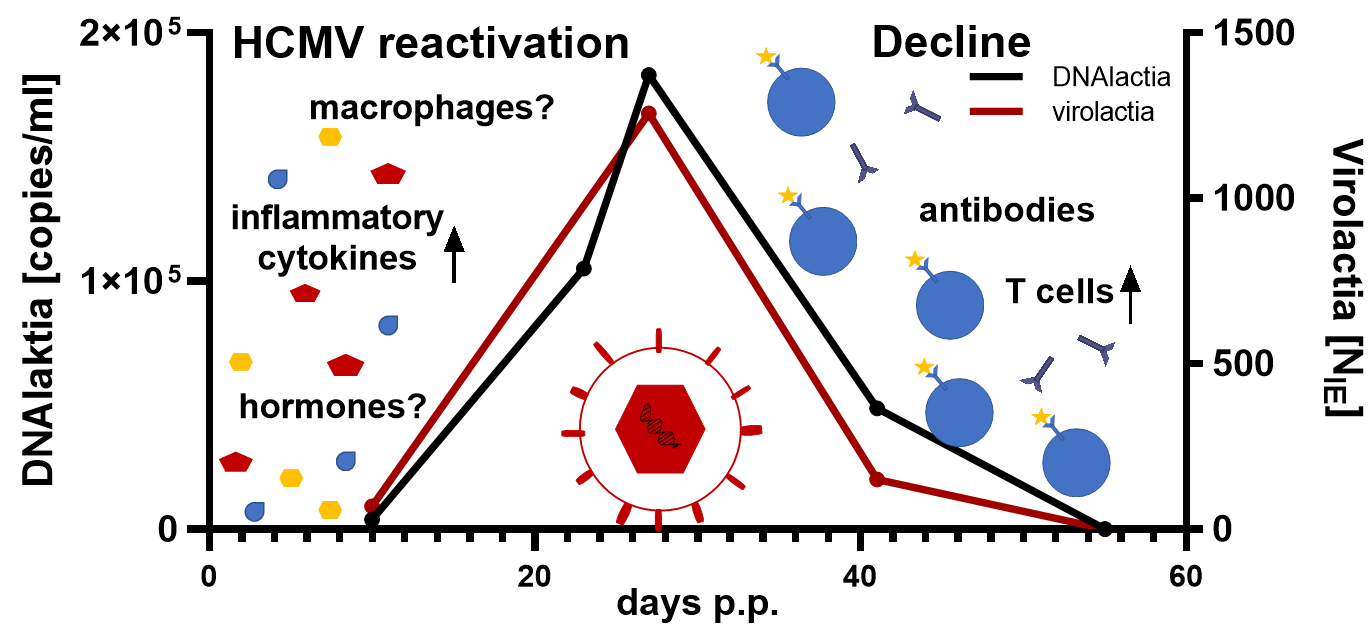


**Supplementary Figure 4.** Hypothesis of HCMV reactivation pattern in breast milk/ in the mammary gland of HCMV-seropositive mothers. HCMV reactivation might be triggered by hormones and inflammatory cytokines leading dormant HCMV in monocytes to reactivate while morphing into macrophages. The unimodal course might be decreasing due to antibodies and T cell activities.

**Supplementary Table 1:** Antibodies and tetramers

| **BlooMil antibodies** | **Conjugate** | **Catalogue number** | **Clone** | **Company** |
| --- | --- | --- | --- | --- |
| Mouse Anti-Human cluster of differentiation (CD) 3 | Alexa Fluor® (A) 700 | # 557943 | UCHT1 | BD Pharmingen^TM^ |
| Anti-human CD4 antibody | Brilliant Violet (BV) 605™ | # 317438 | Okt 04 | Biolegend |
| Mouse anti-Human CD8 | Allophycocyanine (APC)-H7 | # 560179 | SK1 | BD Pharmingen^TM^ |
| Anti-human CD14 antibody | Phycoerythrin (PE)-Cy7 | # 301814 | M5E2 | Biolegend |
| Anti-human CD38 antibody | PE | # 356604 | HB-7 |  |
| Anti-human CD45 antibody | BV 421™ | # 304032 | HI30 |  |
| Anti-human CD45RA antibody | APC | # 304112 | HI100 |  |
| Anti-human CD56 (Neural cell adhesion molecule (NCAM)) antibody | Fluorescein isothiocyanate (FITC) | # 318304 | HCD56 |  |
| Anti-human CD197 (C-C chemokine receptor type 7 (CCR7)) antibody | Biotin | # 353240 | G043H7 |  |
| Streptavidin Brilliant Violet 510™ | BV 510™ | # 405234 | - |  |
| Mouse Anti-Human HLA-DR | Peridinin-Chlorophyll-Protein (PerCP)-Cy™5.5 | # 552764 | G46-6 | BD Pharmingen^TM^ |
| Anti-human HLA-A*02 antibody | PE | # 343305 | BB7.2 | Biolegend |
| HLA-A*02:01 | APC | pp65 (495-504) NLVPMVATV | # HA02-010 (APC) | Tetramer Shop |
| HLA-A*02:01 | PE | pp65 (495-504) NLVPMVATV | # HA02-010 (PE) |  |
| HLA-A*02:01 | APC | IE1 (316-324) VLEETSVML | # HA02-028 (APC) |  |
| HLA-A*02:01 | BV510 | IE1 (316-324) VLEETSVML | # HA02-028 (BV510) |  |

**Supplementary Table 2**: Synopsis of leukocyte subsets in breast milk (BM) and blood. Friedman test shows significances in the kinetics, while the linear mixed model points out differences in the development of the kinetics between HCMV-seropositive or- negative mothers.

| **Cell subsets** | **mate-rial** | **CMV** | **T1 [% mean ± SD]** | **T2 [% mean ± SD]** | **T3 [% mean ± SD]** | **T4 [% mean ± SD]** | **Fried-man test [p-value]** | **linear mixed model** |
| --- | --- | --- | --- | --- | --- | --- | --- | --- |
| **CD45^+^ Leukocytes** | blood | IgG+ | **86.6 ± 5.8** | **91.3 ± 3.3** | **90.9 ± 3.1** | **92.0 ± 3.7** | **0.001** | 0.280 |
|  |  | IgG- | **78.0 ± 26.2** | **90.7 ± 3.3** | **92.3 ± 1.7** | **92.0 ± 4.0** | **0.004** |  |
|  | BM | IgG+ | 8.0 ± 16.4 | 6.6 ± 7.4 | 9.50 ± 17.5 | 4.09 ± 5.0 | 0.208 | 0.537 |
|  |  | IgG- | 3.7 ± 7.1 | 3.6 ± 5.4 | 7.7 ± 12.4 | 5.6 ± 7.9 | 0.197 |  |
|  | | | | | | | |  |
| **CD14^+^ Monocytes** | blood | IgG+ | 18.6 ± 7.5 | 16.0 ± 3.9 | 18.1 ± 4.9 | 17.2 ± 4.9 | 0.196 | 0.437 |
|  |  | IgG- | 18.8 ± 4.9 | 18.3 ± 3.7 | 18.7 ± 5.8 | 17.4 ± 5.1 | 0.293 |  |
|  | BM | IgG+ | **27.0 ± 11.7** | **22.6 ± 12.7** | **12.3 ± 5.3** | **15.8 ± 11.0** | **0.004** | 0.212 |
|  |  | IgG- | **25.1 ± 17.4** | **18.4 ± 12.4** | **16.6 ± 10.6** | **15.9 ± 8.8** | **0.045** |  |
|  | | | | | | | |  |
| **CD14^+^, HLA-DR^+^ MDSC** | blood | IgG+ | 17.5 ± 7.1 | 14.9 ± 3.6 | 17.1 ± 4.5 | 16.3 ± 4.7 | 0.078 | 0.341 |
|  |  | IgG- | 17.7 ± 4.6 | 17.3 ± 3.5 | 17.7 ± 5.7 | 16.3 ± 4.8 | 0.430 |  |
|  | BM | IgG+ | **3.1 ± 3.1** | **2.6 ± 3.5** | **1.1 ± 0.8** | **1.0 ± 0.8** | **0.009** | 0.585 |
|  |  | IgG- | 6.0 ± 9.6 | 2.6 ± 2.3 | 1.9 ± 1.3 | 1.9 ± 1.5 | 0.672 |  |
|  | | | | | | | |  |
| **CD3^+^ T cells** | blood | IgG+ | 57.3 ± 9.7 | 60.3 ± 6.5 | 57.6 ± 7.9 | 59.7 ± 5.9 | 0.514 | 0.829 |
|  |  | IgG- | 58.1 ± 5.4 | 59.5 ± 4.9 | 58.9 ± 7.9 | 59.6 ± 7.1 | 0.160 |  |
|  | BM | IgG+ | **25.1 ± 14.4** | **29.7 ± 18.5** | **38.6 ± 19.7** | **43.7 ± 15.1** | **0.008** | 0.043 |
|  |  | IgG- | 25.6 ± 10.2 | 25.6 ± 11.5 | 24.6 ± 11.4 | 21.9 ± 13.3 | 0.218 |  |
|  | | | | | | | |  |
| **CD4^+^ T cells** | blood | IgG+ | **62.6 ± 7.5** | **62.0 ± 7.1** | **61.6 ± 7.8** | **60.6 ± 7.5** | **0.015** | 0.901 |
|  |  | IgG- | 64.3 ± 5.6 | 64.2 ± 5.6 | 63.5 ± 6.1 | 63.1 ± 6.2 | 0.564 |  |
|  | BM | IgG+ | 51.8 ± 9.3 | 49.9 ± 10.5 | 50.6 ± 12.3 | 49.4 ± 9.1 | 0.863 | 0.141 |
|  |  | IgG- | 60.3 ± 7.0 | 61.0 ± 11.9 | 63.3 ± 10.0 | 65.9 ± 6.5 | 0.077 |  |
|  | | | | | | | |  |
| **CD8^+^ T cells** | blood | IgG+ | 30.1 ± 6.7 | 30.7 ± 6.2 | 30.9 ± 6.9 | 31.5 ± 6.5 | 0.089 | 0.854 |
|  |  | IgG- | 29.2 ± 3.9 | 29.3 ± 4.1 | 29.6 ± 4.5 | 29.9 ± 4.6 | 0.668 |  |
|  | BM | IgG+ | 33.7 ± 6.0 | 36.6 ± 8.9 | 36.5 ± 10.5 | 37.5 ± 6.9 | 0.184 | 0.028 |
|  |  | IgG- | 27.1 ± 6.6 | 26.2 ± 7.1 | 24.9 ± 8.1 | 22.7 ± 6.4 | 0.356 |  |
|  | | | | | | | |  |
| **CD4^+^ CD38^+^ T cells** | blood | IgG+ | **67.4 ± 10.1** | **66.2 ± 9.2** | **65.0 ± 10.1** | **63.8 ± 10.0** | **0.006** | 0.288 |
|  |  | IgG- | 67.4 ± 10.9 | 68.5 ± 11.3 | 67.9 ± 9.5 | 67.9 ± 9.8 | 0.840 |  |
|  | BM | IgG+ | 42.0 ± 12.1 | 47.0 ± 13.7 | 46.9 ± 11.7 | 45.0 ± 9.6 | 0.255 | 0.349 |
|  |  | IgG- | 41.5 ± 16.0 | 41.3 ± 10.5 | 36.5 ± 6.9 | 34.7 ± 7.0 | 0.293 |  |
|  | | | | | | | |  |
| **CD8^+^ CD38^+^**  **T cells** | blood | IgG+ | **69.8 ± 10.7** | **67.7 ± 9.7** | **65.9 ± 10.1** | **64.1 ± 10.9** | **<0.001** | 0.501 |
|  |  | IgG- | 66.7 ± 12.5 | 67.3 ± 11.7 | 66.1 ± 11.5 | 64.5 ± 11.2 | 0.696 |  |
|  | BM | IgG+ | 56.8 ± 9.0 | 57.9 ± 11.6 | 63.0 ± 12.0 | 61.8 ± 10.6 | 0.083 | 0.603 |
|  |  | IgG- | 59.4 ± 12.8 | 59.5 ± 12.0 | 60.2 ± 11.1 | 58.2 ± 13.0 | 0.696 |  |
|  | | | | | | | |  |
| **CD4^+^ HLA-DR^+^ T cells** | blood | IgG+ | 2.3 ± 1.4 | 2.7 ± 1.4 | 3.0 ± 2.0 | 2.7 ± 1.3 | 0.057 | 0.442 |
|  |  | IgG- | 2.1 ± 1.2 | 2.0 ± 0.8 | 1.9 ± 0.5 | 1.6 ± 0.4 | 0.451 |  |
|  | BM | IgG+ | **11.1 ± 4.4** | **14.9 ± 6.8** | **18.6 ± 7.2** | **19.6 ± 7.3** | **<0.001** | 0.108 |
|  |  | IgG- | 15.1 ± 10.4 | 15.1 ± 4.2 | 15.4 ± 5.7 | 14.8 ± 7.0 | 0.472 |  |
|  | | | | | | | |  |
| **CD8^+^ HLA-DR^+^ T cells** | blood | IgG+ | 7.1 ± 3.6 | 6.4 ± 2.8 | 6.2 ± 3.0 | 5.4 ± 2.8 | 0.089 | 0.378 |
|  |  | IgG- | 2.5 ± 1.2 | 3.0 ± 1.3 | 3.4 ± 1.6 | 2.6 ± 1.5 | 0.615 |  |
|  | BM | IgG+ | **15.3 ± 8.1** | **18.3 ± 9.1** | **24.9 ± 12.5** | **25.1 ± 9.2** | **0.001** | 0.327 |
|  |  | IgG- | 9.8 ± 4.1 | 15.0 ± 6.4 | 16.8 ± 7.5 | 15.1 ± 9.0 | 0.494 |  |
|  | | | | | | | |  |
| **CD4^+^ T_N_** | blood | IgG+ | **56.5 ± 10.6** | **54.1 ± 9.9** | **53.3 ± 11.0** | **51.7 ± 10.5** | **0.008** | 0.025 |
|  |  | IgG- | 60.0 ± 12.2 | 62.7 ± 12.3 | 61.6 ± 10.0 | 61.5 ± 10.7 | 0.293 |  |
|  | BM | IgG+ | **3.8 ± 2.0** | **4.1 ± 1.9** | **3.9 ± 3.0** | **2.6 ± 1.3** | **0.015** | 0.349 |
|  |  | IgG- | 4.0 ± 1.8 | 4.0 ± 1.8 | 3.6 ± 1.3 | 3.9 ± 2.0 | 0.540 |  |
|  | | | | | | | |  |
| **CD8^+^ T_N_** | blood | IgG+ | **51.6 ± 11.7** | **48.9 ± 11.9** | **47.4 ± 12.4** | **45.2 ± 12.6** | **<0.001** | 0.043 |
|  |  | IgG- | 63.2 ± 10.7 | 66.3 ± 10.6 | 63.5 ± 9.2 | 62.7 ± 11.1 | 0.356 |  |
|  | BM | IgG+ | **15.8 ± 5.0** | **15.3 ± 5.9** | **13.1 ± 6.1** | **10.6 ± 4.3** | **0.001** | 0.379 |
|  |  | IgG- | 13.7 ± 6.4 | 12.5 ± 4.7 | 13.4 ± 5.6 | 11.7 ± 4.0 | 0.323 |  |
|  | | | | | | | |  |
| **CD4^+^ T_CM_** | blood | IgG+ | 30.1 ± 7.2 | 30.9 ± 7.6 | 31.2 ± 7.5 | 31.2 ± 6.3 | 0.500 | 0.242 |
|  |  | IgG- | 31.5 ± 11.7 | 29.6 ± 11.2 | 29.7 ± 8.8 | 30.1 ± 8.9 | 0.339 |  |
|  | BM | IgG+ | **55.0 ± 7.0** | **54.4 ± 7.9** | **52.3 ± 9.6** | **46.8 ± 7.9** | **0.002** | 0.295 |
|  |  | IgG- | 51.0 ± 13.4 | 52.7 ± 9.6 | 51.9 ± 10.9 | 50.9 ± 10.2 | 0.896 |  |
|  | | | | | | | |  |
| **CD8^+^ T_CM_** | blood | IgG+ | 5.4 ± 2.8 | 5.2 ± 2.8 | 5.1 ± 2.5 | 5.3 ± 2.1 | 0.392 | 0.858 |
|  |  | IgG- | 4.9 ± 2.5 | 4.3 ± 2.0 | 4.3± 1.8 | 4.7 ± 1.8 | 0.266 |  |
|  | BM | IgG+ | 11.6 ± 5.1 | 10.7 ± 3.7 | 11.3 ± 5.0 | 10.7 ± 4.5 | 0.678 | 0.740 |
|  |  | IgG- | 12.1 ± 5.3 | 11.5 ± 5.4 | 13.8 ± 7.6 | 12.3 ± 5.0 | 0.589 |  |
|  | | | | | | |  |  |
| **CD4^+^ T_EM_** | blood | IgG+ | **10.5 ± 6.3** | **11.5 ± 5.4** | **12.0 ± 5.6** | **13.6 ± 7.1** | **<0.001** | 0.053 |
|  |  | IgG- | **7.8 ± 1.7** | **6.9 ± 1.3** | **7.9 ± 1.9** | **7.7 ± 2.0** | **0.036** |  |
|  | BM | IgG+ | **38.4 ± 6.4** | **39.1 ± 7.8** | **41.6 ± 8.6** | **47.9 ± 7.6** | **<0.001** | 0.237 |
|  |  | IgG- | 41.4 ± 13.1 | 40.6 ± 10.6 | 41.8 ± 10.7 | 42.3 ± 10.6 | 0.923 |  |
|  | | | | | | | |  |
| **CD8^+^ T_EM_** | blood | IgG+ | **10.3 ± 4.1** | **11.1 ± 5.0** | **11.8 ± 5.2** | **12.8 ± 5.4** | **<0.001** | 0.106 |
|  |  | IgG- | 10.0 ± 5.3 | 8.7 ± 4.1 | 9.7 ± 4.0 | 10.6 ± 5.0 | 0.104 |  |
|  | BM | IgG+ | **30.0 ± 8.3** | **30.0 ± 10.9** | **33.0 ± 9.6** | **35.8 ± 11.1** | **0.027** | 0.448 |
|  |  | IgG- | **28.5 ± 10.0** | **32.3 ± 12.7** | **31.7 ± 10.9** | **34.9 ± 12.7** | **0.026** |  |
|  | | | | | | | |  |
| **CD4^+^ T_EMRA_** | blood | IgG+ | 3.0 ± 2.2 | 3.6 ± 3.0 | 3.4 ± 2.6 | 3.6 ± 2.6 | 0.295 | 0.421 |
|  |  | IgG- | 0.7 ± 0.3 | 0.8 ± 0.3 | 0.8 ± 0.3 | 0.7 ± 0.3 | 0.218 |  |
|  | BM | IgG+ | 2.7 ± 2.2 | 2.4 ± 1.5 | 2.2 ± 1.6 | 2.6 ± 2.5 | 0.295 | 0.668 |
|  |  | IgG- | 3.6 ± 1.9 | 2.7 ± 2.1 | 2.7 ± 1.8 | 3.0 ± 2.4 | 0.253 |  |
|  | | | | | | | |  |
| **CD8^+^ T_EMRA_** | blood | IgG+ | 32.7 ± 9.9 | 34.8 ± 10.1 | 35.7 ± 10.2 | 36.8 ± 11.2 | 0.061 | 0.237 |
|  |  | IgG- | 21.9 ± 6.4 | 20.7 ± 7.1 | 22.5 ± 6.5 | 22.1 ± 7.8 | 0.293 |  |
|  | BM | IgG+ | 42.6 ± 10.8 | 44.1 ± 12.0 | 42.6 ± 11.0 | 42.9 ± 11.5 | 0.392 | 0.529 |
|  |  | IgG- | 45.7 ± 8.9 | 43.7 ± 14.7 | 41.0 ± 13.6 | 41.2 ± 13.6 | 0.753 |  |
|  | | | | | | | |  |
| **NK cells** | blood | IgG+ | 8.9 ± 3.8 | 9.1 ± 3.8 | 9.3 ± 4.2 | 9.4 ± 4.1 | 0.957 | 0.698 |
|  |  | IgG- | 10.8 ± 2.6 | 9.8 ± 2.0 | 9.9 ± 2.4 | 10.2 ± 3.2 | 0.615 |  |
|  | BM | IgG+ | **4.7 ± 3.2** | **3.5 ± 2.2** | **4.1 ± 2.0** | **5.0 ± 3.0** | **0.028** | 0.292 |
|  |  | IgG- | 6.3 ± 2.3 | 5.9 ± 3.5 | 6.2 ± 5.4 | 5.3 ± 3.5 | 0.229 |  |
|  | | | | | | | |  |
| **CD56^+^ T cells** | blood | IgG+ | 5.3 ± 3.6 | 5.3 ± 3.5 | 5.0 ± 3.3 | 5.1 ± 3.2 | 0.248 | 0.420 |
|  |  | IgG- | 4.6 ± 2.7 | 4.1 ± 3.2 | 4.3 ± 3.2 | 4.4 ± 3.2 | 0.292 |  |
|  | BM | IgG+ | **3.6 ± 2.3** | **3.8 ± 3.5** | **3.0 ± 2.0** | **2.8 ± 1.7** | **0.025** | 0.911 |
|  |  | IgG- | 5.1 ± 2.7 | 4.7 ± 3.4 | 3.9 ± 2.7 | 3.8 ± 2.3 | 0.218 |  |
